# Supplementary material for: Surface-Attached Model Lipid Membranes Derived from Human Red Blood Cells
Source: Langmuir. 2026 Jan 7;42(2):1947–58. doi: 10.1021/acs.langmuir.5c04351 (PMC12825389; doi:10.1021/acs.langmuir.5c04351)
Supplement: Supplementary file 1 [file la5c04351_si_001.pdf]

Supporting Information

Surface-attached model lipid membranes derived from human red blood cells

Authors

Sanyukta Prakash Mudakannavar**, Matthew D. Mitchell**, Katherine Bai, Robert J. Rawle*

Affiliation

Department of Chemistry, Williams College, Williamstown, MA, 01267, USA

**Co-first author contribution

*Corresponding author email: bjr2@williams.edu

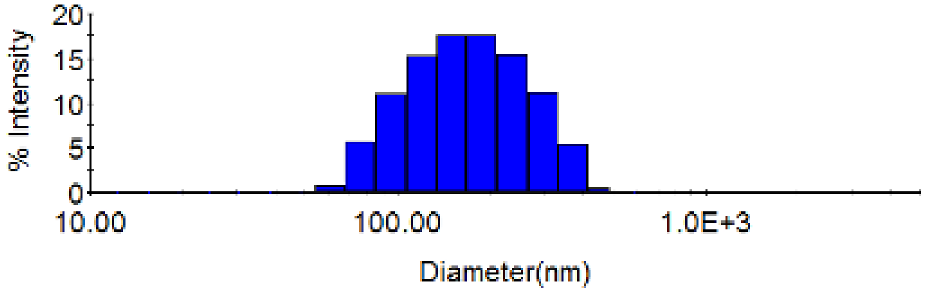

Figure S1. Dynamic light scattering characterization of labeled RBC liposomes. *Mean diameter = 198 nm.*

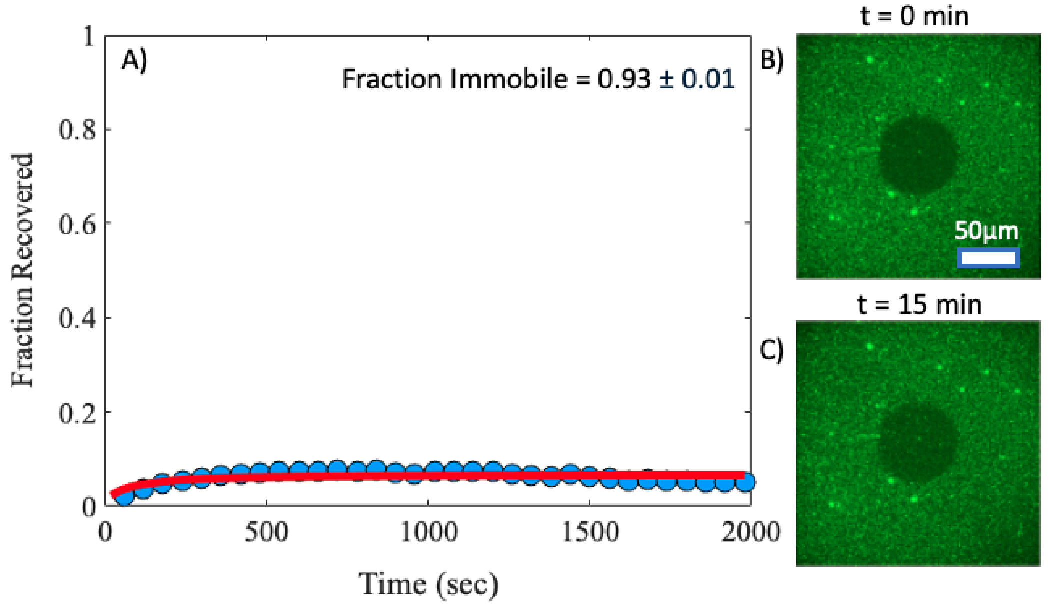

Figure S2. SLBs formed from RBC liposomes alone exhibit lack of lipid mobility. *RBC liposomes labeled with Oregon Green-DHPE were deposited on a glass coverslip to form an SLB. Lipid mobility was assessed by FRAP of the OG-DHPE. A) Example FRAP recovery curve (blue circles = data, red line = fit to FRAP diffusion model,* ***Equation 2*** *in the main text). Fraction recovered is the normalized fluorescence intensity within the photobleached spot, background corrected for residual photobleaching that occurred during the time-lapse imaging. Fraction recovered = 1 was set to the fluorescence intensity immediately prior to photobleaching. Fraction recovered = 0 was set to the fluorescence intensity immediately after photobleaching (t = 0). The immobile fraction calculated from the model fit is shown as the average ± standard deviation of 5 sample replicates. B) and C) Example fluorescence micrographs at t = 0 and t = 15 min, respectively.*

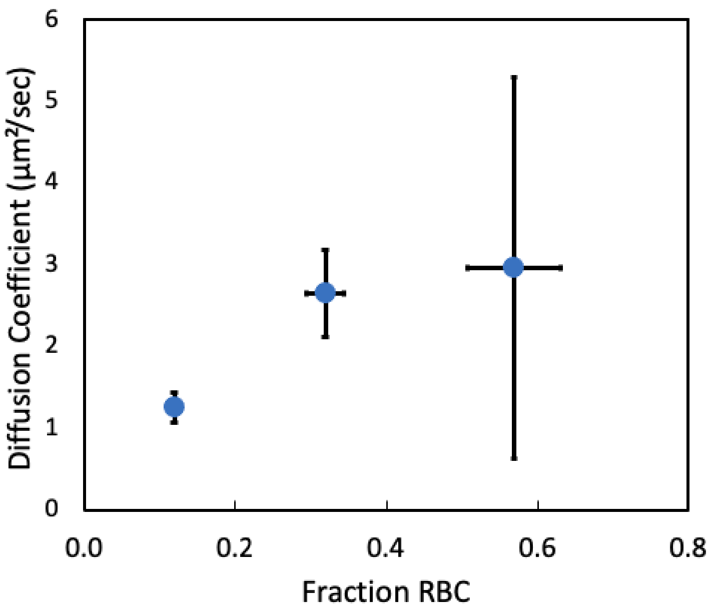

***Figure S3. Plot of estimated diffusion coefficients versus the fraction of SLB composed of the RBC liposomes (Fraction RBC)**. Diffusion coefficients were determined by FRAP diffusion model fits (values shown are average ± standard deviation of 3 sample replicates). Fraction RBC was determined by total image fluorescence comparisons to a standard SLB composed only of rupture vesicles (see main text and Materials and Methods for details). Values shown are average ± propagated error of standard deviations of experimental and standard SLB samples. Standard deviations were calculated from ≥ 20 image locations across 2 sample replicates. Note that the immobile fraction also increases with Fraction RBC (compare to main text **Figure 3D**). As the immobile fraction increases, the diffusion coefficient estimation becomes less precise and can be less accurate if inhomogeneous fluorescence recovery occurs within the photobleached region.*

Table S1. Lipid diffusion coefficients of RBC-SLBs compared to SLBs from prior literature

**Membrane Type**	**Diffusion coefficient ($\mu m^2/sec$)**	**Data Source**
RBC-SLBs	1.3 to 3	This Report
Mitochondrial membrane mimic SLB (48/28/10/4 PC/PE/PI/DOPS/CL)	1.6 to 3.4	*Ref 23*
Bacterial outer membrane vesicle SLBs (+ PEGylated rupture vesicles)	0.28 to 0.36	*Ref 10*
Cell plasma membrane bleb SLB (+ PEGylated rupture vesicles)	0.30 ± 0.03	*Ref 9*
Pure DOPC SLB	3.1 ± 0.1	*Ref 24*

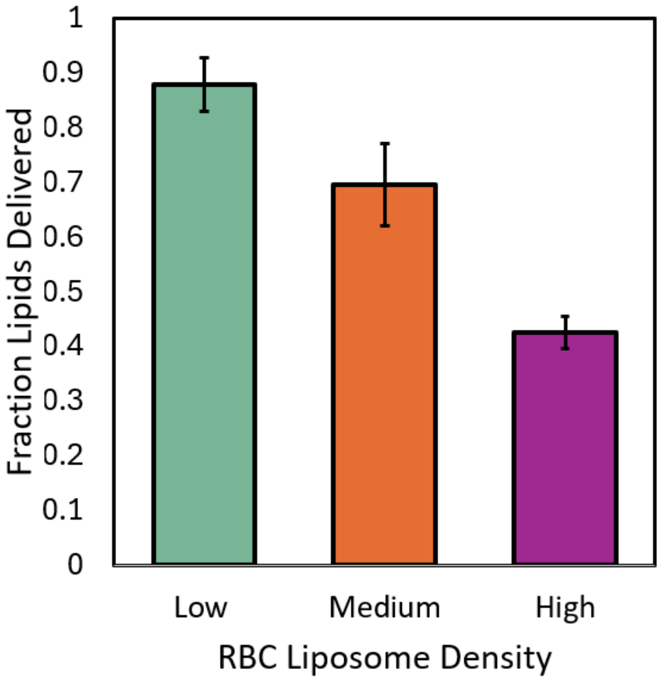

Figure S4. Fraction of RBC lipids delivered to RBC-SLBs prepared with different densities of RBC liposomes. *RBC-SLBs were prepared using the rupture vesicle strategy, but with varying initial densities of labeled RBC liposomes (Low = $Fraction_{RBC}$ ~0.1, Medium = $Fraction_{RBC}$ ~0.3, High = $Fraction_{RBC}$ ~0.6. Fraction of Lipids Delivered was calculated by dividing the total fluorescence in each SLB image after excluding any punctate spots (assumed to be unmerged or incompletely merged liposomes) by the total fluorescence in each image, including the spots. Values shown are average ± standard deviation of 3 sample replicates.*

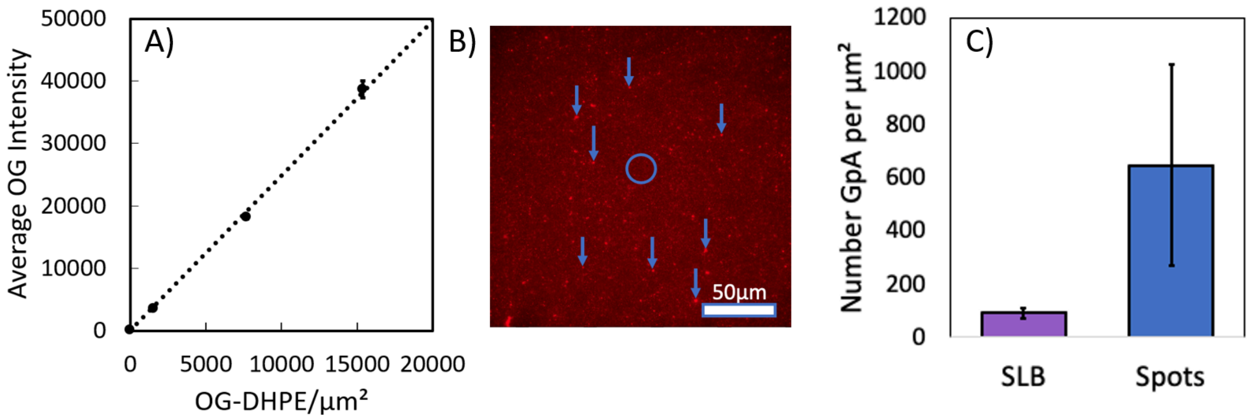

Figure S5. Surface density of glycophorin A (GpA) in different regions of RBC-SLBs. *A) shows a calibration curve of surface density of Oregon Green-DHPE (OG-DHPE) lipid in SLBs versus measured Oregon Green intensity in fluorescence micrographs. SLBs were prepared using synthetic liposomes (no RBCs) containing a known amount of OG-DHPE lipid, ranging from 0 to 0.5 mol%. The surface density of OG-DHPE was calculated using a published cross-sectional area (65 Å^2, Ref 42) of POPC, the predominant lipid in the SLB (≥ 99 mol%). Full details in the Materials and Methods. Values shown are average ± standard deviation of ≥ 6 image locations in each sample. B) Fluorescence micrograph of RBC-SLB immunofluorescently labeled for GpA. RBC-SLB was formed using the rupture vesicle approach, and labeled with primary antibody = mouse IgG anti-glycophorin A (CD235a), secondary antibody = goat anti-mouse IgG with Alexa 647. The circled region denotes an example area of homogeneous fluorescence in the SLB, whereas the arrows indicate examples of clustered spots. C) Bar graph of calculated surface density of GpA in different SLB regions. Fluorescence micrograph Alexa 647 intensity was converted into GpA surface density using an empirically determined Alexa 647:Oregon Green scaling factor, and then comparing to the OG calibration curve in part A, also accounting for the Alexa 647/antibody ratio. Full details in the Materials and Methods. Values shown are average ± standard deviation of the regions shown in part B.*

Table S2. Total protein concentration of parent RBC ghosts and resulting RBC liposomes measured by BCA assay

**Membrane Type**	**Protein Concentration (µg/mL)[a]**
Parent RBC ghosts	1000 ± 100
Resulting RBC liposomes	93 ± 5

[a] *Values shown are mean ± standard deviation of 3 sample replicates.*

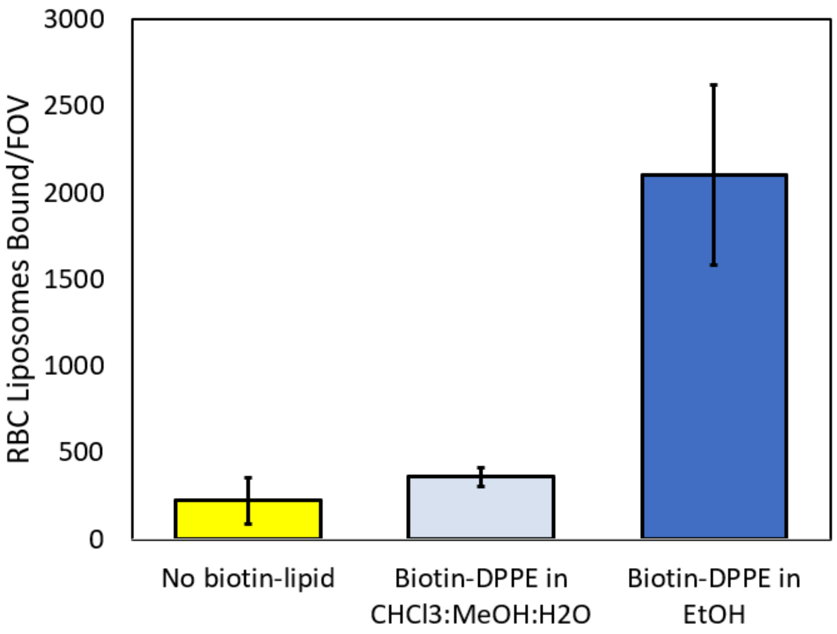

Figure S6. Proper tethering of RBC liposomes requires incorporation of biotin-lipid dissolved in the proper solvent. *Oregon Green-labeled RBC liposomes were prepared either without biotin-DPPE lipid or with biotin-DPPE diluted into two different solvent mixtures – 65/35/8 chloroform/methanol/water (v/v/v, CHCl3:MeOH:H2O) or ethanol (EtOH). The labeled RBC liposomes were then tethered to a polymer supported coverslip as described (see schematic in* ***Figure 7A****). Shown are the number of tethered RBC liposomes per microscope field-of-view (FOV), mean ± standard deviation of ≥8 image areas.*
